# Healthcare-Related Regret among Nurses and Physicians Is Associated with Self-Rated Insomnia Severity: A Cross-Sectional Study

**DOI:** 10.1371/journal.pone.0139770

**Published:** 2015-10-08

**Authors:** Ralph E. Schmidt, Stephane Cullati, Elizabeth Mostofsky, Guy Haller, Thomas Agoritsas, Murray A. Mittleman, Thomas V. Perneger, Delphine S. Courvoisier

**Affiliations:** 1 Department of psychology, University of Geneva, Geneva, Switzerland; 2 Quality of Care Unit, University Hospitals of Geneva, Geneva, Switzerland; 3 Cardiovascular Epidemiology Research Unit, Department of Medicine, Beth Israel Deaconess Medical Center, Harvard Medical School, Boston, Massachusetts, United States of America; 4 Department of Epidemiology, Harvard School of Public Health, Boston, Massachusetts, United States of America; 5 Department of Anesthesiology, Pharmacology, Intensive Care, Geneva University Hospitals, Geneva, Switzerland; 6 Department of Clinical Epidemiology and Biostatistics, McMaster University, Hamilton, Ontario, Canada; 7 Division of Clinical Epidemiology, University Hospitals of Geneva, Geneva, Switzerland; Oasi Institute for Research and Prevention of Mental Retardation, ITALY

## Abstract

To examine the association between healthcare-related regrets and sleep difficulties among nurses and physicians, we surveyed 240 nurses and 220 physicians at the University Hospitals of Geneva. Regret intensity and regret coping were measured using validated scales. Sleep difficulties were measured using the Insomnia Severity Index (ISI), and an additional question assessed the frequency of sleeping pill use. After controlling for sex, profession, years of experience, rate of employment, and depression as well as for all other regret-related variables, the following variables remained significantly associated with self-rated severity of insomnia: regret intensity (slope = 1.32, p = 0.007, 95%CI: [0.36; 2.29], std. coefficient = 0.16) and maladaptive (e.g., rumination) emotion-focused coping (slope = 1.57, p = 0.002, 95%CI: [0.60; 2.55], std. coefficient = 0.17) remained significant predictors of self-rated insomnia severity. If these cross-sectional associations represent causal effects, the development of regret-management programs may represent a promising approach to mitigating sleep difficulties of healthcare professionals.

## Introduction

Sleep difficulties and sleep loss among healthcare providers [1–8] reduce their cognitive abilities [3, 5, 6], and psychomotor performance [4], which can cause medical errors [7]. This empirical evidence concurs with healthcare providers’ self-perception that poor sleep may lead to critical errors at work. In a survey of 150 nurses, 63.5% reported concerns that sleepiness would result in mistakes, and 58.1% worried that sleepiness would cause them to miss changes in patients’ condition [9].

Furthermore, sleep problems are frequent among hospital nurses and physicians. Several studies using the Pittsburgh Sleep Quality Index (PSQI) have shown that around 70% of nurses [9, 10] and 70% of physicians with burnout report poor sleep [11]. Using DSM-IV criteria, one study reported that 18.8%% of physicians suffered from insomnia [9, 11].

While sleep is an important problem among healthcare providers [12], the specific causes of poor sleep in this population are complex. Indeed, they are likely numerous and inter-related. Often cited causes of poor sleep among healthcare professionals include night shift work and number of hours worked [2, 9, 13–16]. The associations of sleep problems with healthcare professionals’ psychological and emotional state [9] and with work environment and job strain [17, 18] have also been investigated.

However, to the best of our knowledge, no study has as yet evaluated whether common (non pathological) emotional reactions in healthcare settings, such as regrets, may impact sleep difficulties. Regret may be defined as an affective, cognitive and physiological state following an experience in which one feels that an outcome would have been better if one had acted differently [19]. This emotion is especially likely to occur in medical settings, where healthcare professionals are often required to make speedy and critical decisions on the basis of complex, uncertain, and abundant information [20, 21]. Upon reflection, healthcare decisions and actions are sometimes perceived as inappropriate [22]. Since healthcare professionals are obliged to make a large number of decisions on a daily basis, they are prone to experiencing more regrets than members of other professions. Thus, examining the association between regrets and sleep in healthcare professions is particularly relevant.

The intensity of regret and the strategies used to regulate regret have been associated with poor sleep in both young [23], and older adults [24], and experimental activation of regret has been shown to delay sleep onset [25]. Courvoisier, Merglen [26] proposed that three distinct aspects of regret may influence sleep among healthcare professionals: the intensity of the most important regret, the accumulation of small regrets, and regret regulation strategies.

The aim of the present study was to investigate the association of healthcare-related regret intensity and regret regulation strategies with sleep quality among physicians and nurses. We hypothesized that (a) the intensity of the most important healthcare-related regret, (b) the number of recently experienced regrets, and (c) the use of maladaptive regret regulation strategies (e.g., self-blame) would be associated with sleep problems.

## Methods

### Study design

We conducted a cross-sectional study of physicians and nurses working at the University Hospitals of Geneva, a Swiss public teaching hospital network including acute and primary care, psychiatric and geriatric facilities with approximately 1900 beds. In 2011, we mailed a paper questionnaire to 1650 randomly selected healthcare professionals (825 nurses and 825 physicians), with up to 3 reminders. Questionnaires were anonymous and sent back by postal mail with no return address. The questionnaire included measures of regret, depression, and insomnia severity, as well as questions about demographics and job characteristics. Exclusion criteria were (a) not working with patients for the last 5 years, (b) recent retirement, or (c) not being a physician or a nurse. The study was approved by the Research Ethics Committee of the University Hospitals of Geneva.

### Instruments and measures

#### Regret

Healthcare-related regret intensity was assessed with the 10-item regret intensity scale (RIS–10) [27]. The RIS–10 examines the intensity of regret at the moment of measurement for an event that occurred up to five years in the past using a five-point Likert scale ranging from 'not at all' to 'absolutely'. Respondents are instructed to choose this event based on the intensity of regret at the time of measurement. Healthcare-related regret regulation was assessed with the 15-item regret coping scale (RCS-HCP)[28]. Using a four-point Likert scale ('never or almost never', ‘sometimes’, ‘often’, and 'always or almost always'), the RCS-HCP measures how frequently the respondent uses three types of regret regulation strategies: problem-focused strategies (e.g., talking to colleagues in order to change practices), and emotion-focused strategies, which can be either adaptive (e.g., acceptance of one’s limitations) or maladaptive (e.g., self-blame). The RIS–10 and the RCS-HCP show good psychometric properties, with internal consistency >0.85 (Cronbach alpha) and test-retest reliability >0.70 (Intra-class correlation 2). The validity of these two scales was considered good since the scales were developed based on a qualitative study of physicians and nurses [29], and a panel of experts, including psychologists, one sociologist, and physicians, ascertained that the scales covered all major aspects of regret intensity and regret coping.

We included additional questions about different aspects of the regret-inducing event:

- “How responsible do you feel of this situation?”: answer options ranged from 0 (no responsibility) to 10 (very high responsibility).
- “Did the regret-inducing event imply an error on your part?”: yes or no.
- “How much support did you receive from your superior?”: Answers options ranged from 0 (none) to 10 (high).

In addition, accumulation of regrets was measured using two questions:

- Regret frequency during the last month: “Within these last 30 days, how many situations with patients have you regretted?”
- Regret intensity during the last month: “What is the mean intensity you would give to these situations within the last 30 days?” Answers ranged from 0 (no intensity) to 10 (highest intensity).

#### Sleep

We assessed sleep problems using the Insomnia Severity Index (ISI), which consists of 7 items rated from 0 (none) to 4 (very much), and yields a total score ranging from 0 to 28. This measure is an index of sleep problems (difficulties initiating and maintaining sleep, early awakenings, satisfaction with current sleep patterns) and the consequences of these problems (interference with daytime functioning, noticeability of impairment to significant others, level of distress caused by the sleep problems) over a one-month interval [30, 31]. The internal consistency of the ISI is at least 0.90 in both a clinical and community samples [32]. Concurrent validity of the ISI has also been demonstrated with other-administered versions of this instrument, polysomnography, and cardiovascular measures [32, 33]. Using a cutoff score of 10 to classify individuals in a community sample as having insomnia (as evaluated by a clinical interview), the ISI has a sensitivity of 86.1% and a specificity of 87.7% [32]. In addition to the ISI, one question examined the frequency of sleeping pill use on a four-point Likert scale (0- ‘not within the last month’, 1- ‘less than once a week’, 2- ‘once or twice per week’', and 3- ‘3 or 4 times a week or more’). Answers were dichotomized (not within the last month vs. once or more often per month).

#### Depression

Depressive symptoms over the last 7 days were measured using the Center for Epidemiologic Studies Depression Scale (CESD–10), a 10-item scale for measuring depressive symptoms in the general population. The measure has been shown to possess good internal consistency (α = 0.85) [34, 35].

Since regret and sleep may be related to professional and demographic characteristics, we also collected information on factors that may act as confounding variables, including sex, profession, years of experience, and rate of employment.

### Analyses

#### Sample size

To accurately estimate regression coefficients, there should be at least 5 times more events (in this study sleeping pill use) than predictors [36], yielding a minimum of 50 events for our 10 predictors (regret variables and confounders included). Based on an expected proportion of healthcare professionals using sleeping pills of 12%, the required sample size is 417 (50/0.12).

#### Statistical analysis

We used multiple linear regression to estimate slopes and 95% confidence intervals for the association between aspects of regret and insomnia severity as assessed by the continuous ISI score. We used multivariable logistic regression to estimate the odds ratios and 95% confidence intervals for the association between aspects of regret and sleeping pill use. For each aspect of regret, we present the results of uni- and multivariate models adjusting for sex, profession, years of experience, rate of employment, and CES-D score. The covariates used for adjustment were chosen to control for confounding effects. For instance, depression could cause sleep problems and change strategies of regret coping, resulting in a biased estimation of the association between regret and sleep. In addition, we constructed a multivariate linear regression model including all aspects of regret and the covariates listed above. The importance of the influence of the regret variables over and above the covariates was estimated with the R^2^ change coefficient of determination statistic.

Since, in our previous analysis, regret intensity and regret coping were on average similar across professions [27, 28], we analyzed the association between regret and sleep for the whole sample. However, as a sensitivity analysis, we also ran the analyses separately for each profession (i.e., not adjusting for profession in the model). Results were very similar across nurses and physicians (data not shown).

We plotted the proportion of respondents reporting sleeping pill use more than once in the past month according to the score for regret intensity and the score for maladaptive coping strategies. Statistical analyses were performed using R (v 3.0.1) (R Core Team, 2013).

## Results

Overall, 240 nurses and 220 physicians returned the survey (31.2% of the 1474 eligible participants). Respondents were on average 39.5 (SD 9.1) years old, and 69.3% were female. Compared to the physicians who participated in the study, the nurses tended to be older and reported more sleep problems and also more frequent use of sleeping pills (Table 1). Among the respondents, 19 (4.1%) did not answer the sleep, regret or depression scales, and were excluded from the analysis.

**Table 1:** Baseline characteristics of 460 healthcare providers at University Hospitals of Geneva, Switzerland, 2011.

		Nurse		Physician	
Characteristics	Categories	N = 240		N = 220	
		N	%	N	%
*Socio-professional*					
Sex	Male	45	18.8	96	43.6
	Female	195	81.3	124	56.4
Age	<30	25	10.4	28	12.8
	30–39	84	35.0	126	57.8
	40–49	76	31.7	45	20.6
	>50	55	22.9	19	8.7
Professional status	Nurse / resident	217	91.6	106	48.4
	Head nurse / Board certified	20	8.4	113	51.6
Rate of employment	0–50%	12	5.0	10	4.6
	51–80%	100	41.7	27	12.4
	81–100%	128	53.3	180	82.9
Years of experience	<3	15	6.3	20	9.3
	3–5	8	3.3	54	25.1
	6–10	50	20.9	73	33.9
	11–20	67	28.0	44	20.5
	>20	99	41.4	24	11.2
*Regret*		Mean	SD	Mean	SD
Regret intensity (RIS–10)		1.74	0.65	1.70	0.73
Regret coping	Problem-focused	2.92	0.60	2.94	0.59
(RCS-HCP)	Maladaptive	1.83	0.57	1.86	0.70
	Adaptive	2.64	0.51	2.76	0.59
*Depression*		1.88	0.48	1.84	0.39
*Sleep*					
Insomnia severity index (ISI)		8.60	6.03	5.66	5.33
		N	%	N	%
ISI ≥ 10		102	43.6	48	22.4
Sleeping pill use ≥ once/month		44	18.3	30	13.6

### Insomnia Severity Index

A one-unit increase in the intensity of the most important regret in the last five years (range: 1–5) was associated with almost 3 more points in self-rated insomnia severity (ISI range: 0–28) (Table 2). This corresponds to a standardized coefficient of 0.33 (not shown in Table 2). The number of regret-inducing events and the score for maladaptive regret coping strategies were also associated with higher scores of insomnia severity (Table 2). Conversely, higher scores for problem-focused and adaptive regret coping were associated with lower scores of insomnia severity (standardized coefficient of -0.13 and -0.12 respectively). These associations remained significant and of similar strength even after adjusting for depression and socio-professional variables (Table 2). Both regret intensity and the use of maladaptive strategies showed a substantial association with insomnia severity, as the R^2^ change (between a model with only the covariates and a model with the regret variable and the covariates) for regret intensity was 5.1%, and the R^2^ change for maladaptive strategies was 4.7% (Table 2). Feeling supported by one’s superior when the regretted event occurred was associated with lower insomnia severity, but feeling responsible for a regret-eliciting event or reporting that this event was related to a medical error were not.

**Table 2:** Parameter Estimates and 95% Confidence Intervals from Linear Regression Models to Evaluate the Association Between Measures of Regret and Insomnia Severity Index Score.

	Univariable			Adjusted*			
	slope	p	95% CI	slope	p	95% CI	ΔR^2^
Regret intensity	2.79	<0.001	2.03;3.56	2.05	<0.001	1.31;2.78	5.1
Regret coping							
Problem-focused	-1.31	0.005	-2.24;-0.39	-1.28	<0.001	-2.14;-0.42	0.5
Adaptive	-1.32	0.009	-2.32;-0.33	-0.98	0.04	-1.89;-0.06	0.1
Maladaptive	2.94	<0.001	2.12;3.76	2.36	<0.001	1.56;3.17	4.7
Number of regrets in the last month	0.37	0.002	0.13;0.61	0.25	0.02	0.03;0.47	0.1
Mean intensity of regrets in the last month	0.48	<0.001	0.27;0.69	0.33	<0.001	0.14;0.52	1.3
Responsibility	0.06	0.54	-0.13;0.25	0.10	0.25	-0.07;0.28	0.0
Error	-1.00	0.10	-2.20;0.19	-0.06	0.92	-1.20;1.08	0.0
Superior support	-0.26	0.001	-0.41;-0.10	-0.15	0.04	-0.29;-0.01	0.1
Profession (ref: nurse)	-2.94	<0.001	-4.00;-1.88	-2.06	<0.001	-3.20;-0.92	—

*Adjusted for profession (nurse or physician), sex, years of experience, rate of employment, and CES-D depression score.

In a multivariable model including all aspects of regret (ie. regret intensity, the three regret coping strategies, number and average intensity of regrets in the last month, responsibility, evaluation of the event as an error and superior support), as well as the potential confounders added in the previous multivariable model (i.e., depression, demographics, and job characteristics), only regret intensity (slope = 1.32, p = 0.007, 95%CI: [0.36; 2.29], standardized coefficient = 0.16) and maladaptive regret coping strategies (slope = 1.57, p = 0.002, 95%CI: [0.60; 2.55], standardized coefficient = 0.17) remained statistically significant predictors of insomnia severity.

### Sleeping Pill Use

As expected, respondents who reported using sleeping pills at least once in the prior month also reported more sleep problems (mean ISI score for people using sleeping pills more than once a month: 13.0 (SD = 5.8), mean ISI for people using sleeping pills less than once a month: 6.1 (SD = 5.2), p<0.001).

**Fig 1:**
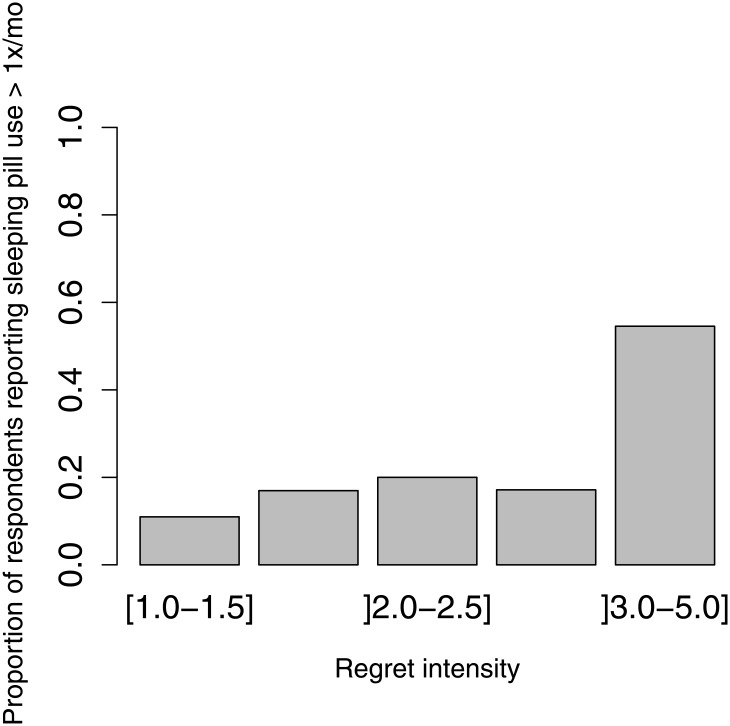
Proportion of respondents who reported using sleeping pills more than once a month according to levels of regret intensity (RIS–10). Levels of regret intensity were aggregated if there were fewer than 10 respondents.

**Fig 2:**
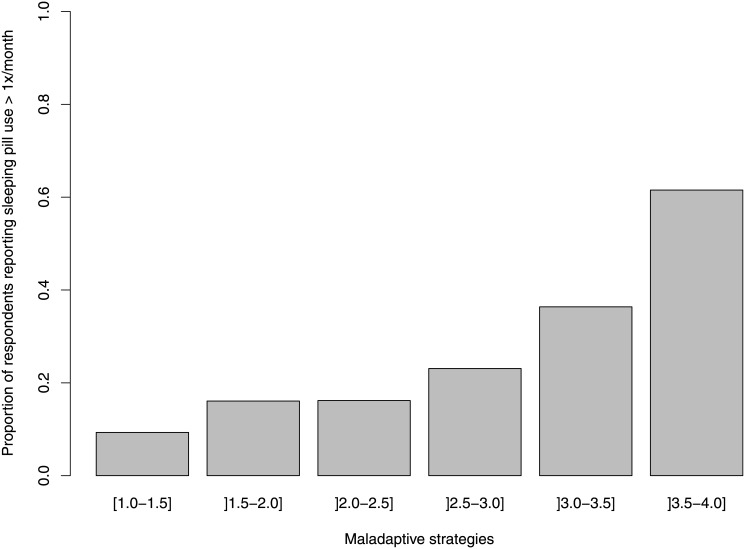
Proportion of respondents who reported using sleeping pills more than once a month by levels of maladaptive strategies.

We observed a strong association between dimensions of regret and whether the healthcare professional used sleeping pills at least once in the prior month (Table 3). For every one unit increase in the regret intensity score, the odds of reporting sleeping pill use was 2.14 times higher (Fig 1); for every one unit increase in the maladaptive regret coping score, the odds of reporting sleeping pill use was 2.25 times higher (Fig 2). These results remained statistically significant event after adjusting for depression, demographics and job characteristics (Table 3). There was no statistically significant association between the use of problem-focused and adaptive strategies and sleeping pill use.

**Table 3:** Odds Ratios and 95% Confidence Intervals from Logistic Regression Models to Evaluate the Association Between Measures of Regret and Sleeping Pill Use.

	Univariable			Adjusted*		
	OR	p	95% CI	OR	p	95% CI
Regret intensity	2.14	<0.001	1.55; 2.97	2.06	<0.001	1.45; 2.94
Regret regulation						
Problem-focused	0.85	0.44	0.56; 1.29	0.76	0.21	0.49; 1.17
Adaptive	0.75	0.23	0.47; 1.19	0.78	0.31	0.49; 1.25
Maladaptive	2.25	<0.001	1.57; 3.25	2.10	<0.001	1.41; 3.14
Number of regrets in the last month	1.16	0.006	1.05; 1.30	1.14	0.02	1.03; 1.28
Mean intensity of regrets in the last month	1.07	0.25	0.96; 1.20	1.01	0.85	0.90; 1.14
Responsibility	1.06	0.21	0.97; 1.15	1.06	0.22	0.97; 1.16
Error	0.99	0.96	0.56; 1.69	1.21	0.52	0.66; 2.17
Superior support	1.00	0.93	0.93; 1.08	1.02	0.64	0.94; 1.09

*Adjusted for profession (nurse or physician), sex, number of years of experience, rate of employment, and CES-D depression score

In multivariate analyses including all aspects of regret, depression, demographics and job characteristics, there was a 1.76 (95%CI: 1.08; 2.92) times higher odds of sleeping pill use associated with a one unit increase in regret intensity and a 1.14 (95%CI 1.01; 1.29) times higher odds of sleeping pill use associated with a one unit increase in the number of regret-inducing events or episodes of regret in the prior month.

## Discussion

In this cross-sectional study of 460 physicians and nurses, healthcare-related regret was associated with higher self-rated insomnia severity and sleeping pill use. The intensity of the most important regret in the past five years, the number of regrets, and the use of maladaptive regret regulation strategies were associated with higher levels of self-reported insomnia severity and higher odds of sleeping pill use. These associations remained statistically significant after adjusting for depression, demographics, and job characteristics. However, neither feeling personally responsible for the regret-eliciting event nor connecting this event to a medical error were associated with sleep problems or sleeping pill use, suggesting that the scope of regrets stretches beyond individual mistakes in medical decisions. In accordance with our proposed theoretical model [26] and our previous qualitative study [29], these results suggest that regret intensity and coping are associated with sleep quality among physicians and nurses. In addition, the findings are consistent with studies indicating that the use of dysfunctional strategies of thought control (e.g., thought suppression) and emotion regulation (e.g., self-blame) may contribute to a state of sleep-interfering cognitive and affective arousal [37]. Other dysfunctional strategies, such as rumination, have been associated with insomnia and more generally with neuroticism [38, 39], which is often higher among individuals with sleep difficulties.

Whereas shiftwork in healthcare settings and its consequences on sleep have been studied extensively [e.g., 2], and pathological emotional states (e.g., burnout [11], anxiety [14]) have also been the focus of much research, there is limited research in recent years on psychological (non pathological) factors associated with sleep in this population. This study suggests that emotion regulation may be strongly associated with healthcare professionals’ sleep, though additional studies are necessary to determine the directionality of the link between regret regulation and sleep. Further research to identify psychological factors and methods of mitigating their adverse consequences are warranted because, unlike work-schedule constraints, they represent a more modifiable risk factor for sleep quality. Modifying work-schedule constraints, for instance to introduce aviation industry work-hour restrictions in the medical system, was estimated at more than $1,000,000 per patient life-year saved [40]. The introduction of such restrictions would also entail an increase of 71% in the US physician workforce and a 174% increase in the number of residents [40].

A promising avenue for future research will be to design interventions that encourage the use of functional regret regulation strategies (i.e., problem-focused strategies; adaptive emotion-focused strategies) and to explore their efficacy in randomized-controlled trials. Regarding the especially maladaptive strategy of self-blame or self-attacking, it has previously been suggested [24] that fostering self-compassion [41] and self-forgiveness [42] might be particularly helpful intervention strategies.

Given the cross-sectional design of this study, causal inferences regarding the effects of regret intensity and regulation on sleep remain speculative. It is not clear whether regret impacts sleep or sleep impacts feelings of regret. For instance, sleep problems could lead to more time for ruminations. However, as mentioned earlier, a recent randomized trial found that experimental activation of regrets at bedtime delays sleep onset [25]. But there is also evidence that sleep loss may in turn undermine emotion regulation: For instance, a longitudinal study of 78 medical residents found that sleep loss intensified negative emotions following daytime disruptive events [43]. Taken together, these different lines of research suggest that the relations between sleep and emotion regulation may best be conceptualized as bi-directional [44]. Specifically, the experience of regrets may interfere with sleep and the resulting sleep loss may increase regret intensity and decrease resources needed to regulate regrets in adaptive ways. Moreover, sleep loss may decrease attentional resources [45], thereby increasing the risk of errors.

Finally, two other limitations of our study warrant discussion. First, only 31.2% of the eligible healthcare professionals responded to the survey, potentially due to the sensitive topic of regrettable behavior in healthcare settings [46]. Therefore, there may be selection bias if survey response was related to sleep habits and regrets. However, similar associations have previously been found in a sample of university students who were obliged to return their questionnaires in fulfillment of a course requirement [23]. It is also of note that response rate did not differ by profession. Second, we did not collect data on anxiety. It is difficult to disentangle the fact that people with higher levels of anxiety may be more likely to experience regret and may also be more likely to experience sleep difficulties. Therefore, anxiety may either confound or mediate the association between regret and sleep quality. However, in our analyses, we adjusted for depressive symptoms, an affective state that is more closely related to regret than anxiety [33, 47], and the associations between regret intensity, maladaptive regret regulation, and sleep problems remained statistically significant. Note that depression may also be part of the potential causal pathway between regret and sleep. The associations reported here may thus be underestimated.

In conclusion, we have shown that regret intensity and the use of maladaptive regret regulation strategies are cross-sectionally associated with sleep problems among health care professionals. Whether regret management can be acted upon, so as to limit its impact on sleep and other negative consequences, is an issue worth exploring.

## Supporting Information

S1 Data Codebook for the data. ID. Provides a unique and anonymous ID. SEX: 1 = male, 2 = female. PROF: nurse or physician. EMPLOYLEV: Rate of employment, 1 = 0–50%, 2 = 50–80%, 3 = 80%-100%. Expcat: Categories of work experience, 1 = <3years, 2 = 3–5 years, 3 = 6–10 years, 4 = 11–20 years, 5 = >20 years. RIS10: regret intensity scale score. PF: problem-focused regret coping strategy score. MA: maladaptive regret coping strategy score. A: adaptive regret coping strategy score. Depression: CES-D score. ISI: insomnia severity index score. Pill: Frequency of pill use in the last month, 0 = <1/month, 1 = ≥ 1/month. RMONTH: number of regret in the last month. RINTEN.MONTH: average intensity of regrets in the last month. RRESPON: “How responsible do you feel of this situation?”:, 0 = no responsibility to 10 = very high responsibility. RERROR: “Did the regret-inducing event imply an error on your part?”, 1 = no, 2 = yes. RSUPPORT: “How much support did you receive from your superior?”, 0 = no support to 10 high support.
(CSV)
